# Potential Lignocellulolytic Microfungi from Pineapple Plantation for Composting Inoculum Additive

**DOI:** 10.1155/2022/9252901

**Published:** 2022-03-24

**Authors:** Bambang Irawan, Inten Wahyuningtias, Niken Ayuningtyas, Ola Apriyani Isky, Salman Farisi, Sumardi Sumardi, Afandi Afandi, Sutopo Hadi

**Affiliations:** ^1^Department of Biology, Faculty of Mathematics and Natural Sciences, The Universitas Lampung, Bandar Lampung 35145, Indonesia; ^2^Department of Soil Science, Faculty of Agriculture, Universitas Lampung Bandar Lampung, Bandar Lampung 35145, Indonesia; ^3^Department of Chemistry, Faculty of Mathematics and Natural Sciences, The Universitas Lampung, Bandar Lampung 35145, Indonesia

## Abstract

Pineapple plantations leave a lot of plant biomass after the planting season ends. The abundant residue of pineapple plants causes problems due to the high content of lignocellulose, which is difficult to decompose naturally. This study aimed to isolate and characterize lignocellulolytic microfungi isolates from pineapple plantations. The information of this study was used as data to prepare an inoculum for the induction of pineapple litter composting that was resistant to stress to pineapple plantation habitat. The results showed that there were 11 dominant lignocellulolytic microfungi isolates found from pineapple litter and plantation soil. Using selective media, the selection showed five cellulolytic (Bioggp 3, 6, 9, 11, and 12); five xylanolytic (Bioggp 3, 6, 8, 9, and 12); and two ligninolytic microfungi isolates (Bioggp 2 and 5). Bioggp 3, 6, 9, and 12 are cellulolytic and xylanolytic with Bioggp 3 showing the highest cellulolytic index (4.0) and xylanolytic index (4.20). Testing of ligninolytic microfungi showed that the Bioggp 5 isolate had a stronger lignin indicator (color intensity = 4.0 and zone ratio of 1.47) than the Bioggp 2 isolate. Bioggp 9 had the highest cellulolytic isolate spore productivity at 4.5 × 10^8^ spores/mL with 93.3% spore viability, and Bioggp 3 had the highest xylanolytic isolate spore productivity at 2.5 × 10^9^ spores/mL with 89.3% spore viability. Bioggp 2 had the highest ligninolytic isolate spore productivity at 1.8 × 10^9^ spores/mL, but Bioggp 5 showed the highest spore viability at 98.0%.

## 1. Introduction

Harvest of pineapple plants will produce huge pineapple leaf waste. Abundant pineapple waste is a problem due to the accumulation of organic waste, which can disrupt the balance of land fertility if it is not decomposed quickly. Pineapple skin contains 23.39% cellulose, 42.72% hemicellulose, and 4.03% lignin [1]. In addition, pineapple humps have 28.53% cellulose, 24.53% hemicellulose, and 5.75% lignin [2].

A cellulose component in pineapple waste is not easily degraded, both chemically and mechanically, because cellulose has crystalline and nonsoluble properties derived from its linear structure [3]. Substantially, lignocelluloses represent the most abundant natural material on Earth, being approximately 50% of all biomass, with an estimated 181.5 billion tonnes produced annually. Of the 8.2 billion tonnes that are currently used, about 7 billion tonnes are produced from dedicated agricultural, grass, and forest land, and another 1.2 billion tonnes stem from agricultural residues [4]. This represents an enormous pool of organic carbon [5]. These waste materials could be turned from liabilities into assets [6, 7]. Organisms utilizing lignocellulosic materials for their carbon and energy sources could be exploited for the conversion of these wastes into products beneficial to man. Therefore, this study is intended to obtain lignocellulolytic microfungi as inducer inoculums capable of decomposing pineapple waste.

Lignocellulolytic microfungi produce lignocellulolytic enzymes, which constitute a large group of mainly extracellular proteins, including ligninolytic enzymes (peroxidases and oxidases) and hydrolytic enzymes (cellulases, hemicellulases, pectinases, chitinases, amylases, proteases, esterases, and mannases) [8]. This study isolated and characterized lignocellulolytic microfungi isolates from pineapple plantations. Characterization was carried out on isolates that had the capability to produce cellulolytic, xylanolytic, and ligninolytic enzymes qualitatively. In addition, each isolate was observed for spore productivity, viability, ability to grow under acidic conditions, temperature above room temperature, and herbicides 1%.

This characterization data are important as the basis for other research for the preparation of additional inoculums to induce composting of pineapple litter. Characters for inoculum selection were based on habitat where compost will be applied. The pineapple plantations in Lampung, Indonesia have acid ultisol soil types, temperatures above 30^ᴏ^C, and herbicide residues. Those factors may interfere with soil fungi growth. Herbicide interference in fungi growth includes suppression of spore germination, inhibition of the rate of linear extension of the mycelia, and abnormalities in growth habits and in patterns of spore production. Also, some herbicides were more fungitoxic [9].

Therefore, it can be assumed the potential fungi for composting inoculum can be selected when they are able to grow under these various stress conditions. The decomposition activity of fungi will presumably continue in the soil when the selected fungi-induced compost is applied to pineapple plantations as biofertlizers.

## 2. Materials and Methods

### 2.1. Materials

**Figure 1:**
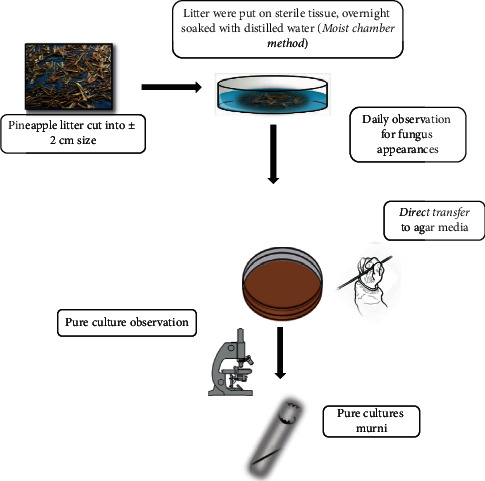
Moist chamber method.

Isolation was carried out using pineapple litter materials taken from pineapple plantations of PT (Great Giant Pineapple, Terbanggi Besar, Lampung, Indonesia). Isolation was carried out by a direct isolation technique combined with a moist chamber method (Figure 1). The moist chamber isolation technique first required that the litter mixture be cut to a size of ±2 cm and then put into a Petri dish containing sterile tissue and distilled water and left to soak for one night, then the distilled water is discarded, but the humidity of the moist chamber must be maintained. Then, observations were made every day to see the fungi that appeared. Each fungus that appeared was taken for its spores by the direct transfer method by touching the tip of the tube that was given, so that the fungal spores that appeared were then transferred to PDA media until pure culture was obtained. The fungal isolates obtained were then coded and, to ensure that there were no similar isolates, the isolates were first observed for their morphology under a microscope [10].

The identification process is carried out by microscopic and macroscopic observations. Macroscopic observations were made by looking at the color of the colony and the surface of the colony. Microscopic observations were carried out on slide culture specimens using the method available in the literature [10]. Identification was performed by looking at the morphology of the fungal isolates based on the identification key and comparing them with book references.

### 2.2. Isolation and Selection of Cellulose-Producing and Degrading Microfungi

Isolation of cellulase-producing microfungi was carried out by the dilution and direct plating methods [10]. The selection of cellulolytic microfungi isolates was performed by a modification of the Congo red method reporter by others [11]. Isolates obtained were cultured in cellulose agar (cellulose 5.0 g, NaNO_3_ 1.0 g, K_2_HPO_4_ 1.8 g, MgSO_4_.7H_2_O 0.09 g, KCl 0.5 g, yeast extract 0.5 g, casein hydrolysate 0.5 g, agar 20 g, and 1 L distilled water). Confirmation of the cellulose-degrading ability of fungal isolates was performed by streaking them on cellulose agar. The bilayer media has been utilized, with PDA (1/5 recipe, agar 1.5 g, and 100 mL of distilled water) as the bottom layer 1.

### 2.3. Isolation and Screening of Xylan-Degrading Microfungi

Isolates obtained were cultured in microfungi xylanolytic medium (1 g xylan from beechwood, 5 g peptone, 5 g yeast extract, 0.2 g K_2_HPO_4_, 20 g agar, and 1000 mL distilled water) [11–14]. Confirmation of the xylan-degrading ability of fungal isolates was performed by streaking on xylan-enriched media. Bilayer media were used, with PDA as the bottom layer (1/5 recipes, agar 1.5, and 100 mL distilled water). The top layer consisted of xylan from 1% beechwood, agar 1.5, and 100 mL distilled water. Once inoculated with microfungi in the middle of the test media, the cultures were then incubated for four days. The media were added with 0.1% Congo red and allowed to stand for 20 min at room temperature. The media were washed with 1 M NaCl (left overnight, if necessary). Isolates producing xylanase formed a halo (clear zone) around the colony. The use of Congo red as an indicator of xylan degradation in an agar medium provides the basis for a rapid and sensitive screening test for xylanolytic microfungi. The xylan-degrading potential of the positive isolates was also qualitatively estimated by calculating the hydrolysis capacity, which is the ratio of the diameter of the clearing zone and the colony [15].

### 2.4. Isolation and Screening of Lignin-Degrading Microfungi

Fungal isolates were isolated on B and K (Boyd & Kohlmeyer) medium (10 g glucose, 2 g peptone, 1 g yeast extract, 18 g agar, and 1 L aquadest) [11–13, 16]. The seven-day-old isolates were then cut into 10 mm diameter sections from the middle of the cultures and transferred to qualitative screening media (guaiacol-supplemented agar containing 10 g glucose, 2 g peptone, 1 g yeast extract, 18 g agar, and 4 mM guaiacol in 1 L water) [17]. The inoculated plates were incubated at 25°C in the dark for seven days. An intense brown color produced under and around the fungal colony was considered as a positive reaction resulting from guaiacol oxidation [18].

### 2.5. Characterization of Lignocellulolytic Microfungi

Characterization includes the ability of fungi to produce spores and their viability at different pH, temperature, and herbicide stress conditions. Spore productivity was calculated from 14-day-old isolates grown on selective media with serial dilution methods. Spore viability was calculated for its CFU value by using the pour-plate technique on PDA. Then, the characterization of isolates was carried out by growing at a low pH of 3, 4 and 5 as well as temperatures above room temperature (30°C, 35°C, and 37°C). In addition, fungal growth was also observed in media with the addition of 1% herbicides (ametryne and diuron). Ametryne, methylthio-s-triazine, is a herbicide with a slower path of degradation and provides longer herbicidal activity. Diuron is the trade name for 3-(3, 4-dichlorophenyl)-1,1-dimethylurea (DCMU), an algicide and herbicide of the arylurea class that inhibits photosynthesis. This herbicide is thought to inhibit the growth of fungi, and it is hoped that fungi that are resistant to it can be obtained.

## 3. Results and Discussion

The results of the isolation and enzymatic activity tests on isolates of pineapple plantation microfungi are shown in Table 1. Isolation using selected media on the substrate of biomass material and soil from pineapple plantations has obtained 11 isolates classified as lignocellulolytic isolates. The fungal enzymatic activity was observed qualitatively by looking at the presence or absence of a clear zone (±), while quantitative activity was observed by looking at the enzymatic index (Tables 2–4).

Several isolates show positive test results indicated by a clear zone formed around the colony as an indication that the isolate produces cellulase enzymes in agar media (Bioggp 3, 6, 9, 11, and 12). Xylanolytic microfungi selection showed that five microfungi isolates (Bioggp 3, 6, 8, 9, and 12) could degrade xylan. Two isolates positively produced enzymes to degrade lignin (Bioggp 2 and 5), shown by the formation of a brown color on the bottom and around the colony. The data also showed that there were a number of isolates that showed two enzymatic abilities at once, namely, cellulolytic and xylanolytic, including Bioggp 3 (*Aspergillus* sp. 1), Bioggp 6 (*Aspergillus* sp. 2), Bioggp 9 (*Penicillium* sp), and Bioggp 12 (*Paecilomyces* sp). Lignocellulolytic microfungi often have various extracellular enzymes because microfungi are able to degrade lignocellulose substrate into simpler components. They have two types of extracellular enzymatic systems: hydrolytic and ligninolytic systems, which are very efficient. Both systems produce cellulase, hemicellulase, and ligninase to degrade polysaccharides and lignin and to open phenyl rings [19].

All isolates showed high spore productivity and viability (Table 2). The data show that Bioggp 9 produced the largest number of spores and CFU at 4.5 × 10^8^ spores/mL and 1.2 × 10^8^ CFU/mL, respectively. However, the highest percentage of fungal viability is found in Bioggp 6 (95.1%), and the lowest is in Bioggp 12 (78.7%). Fungal isolates can be categorized as having high spore viability if they reach a CFU average of ≥1 × 10^7^ CFU/mL. Furthermore, they are also categorized into moderate levels if they have a CFU average of ≥1 × 10^6^ CFU/mL [20]. Thus, the Bioggp 3, 6, and 9 isolates can be categorized into high levels of spore viability, while Bioggp 11 and 12 are included in the moderate category.

The results showed that the average number of spore production and viability in xylanolytic microfungi had varying values (Table 3). The highest numbers of spore production and viability were found in Bioggp 9, which were 4.6 × 10^9^ spores/mL and 2.6 × 10^8^ CFU/mL, respectively. Bioggp 12 had the lowest viability percentage compared with other isolates of the leaf litter of pineapple. The results showed that the viability of the xylanolytic microfungi spores was lower than spore production.

Table 4 presents the number and viability of spore isolates of Bioggp 2 and Bioggp 5 after incubation for 14 days on ligninolytic qualitative testing media enriched with guaiacol. The results showed that Bioggp 2 produced the amount and viability of spores at 1.8 × 10^9^ spores/mL and 6.0 × 10^8^ CFU/mL, respectively, equal to 95%; whereas, Bioggp 5 produced 8.3 × 10^8^ spores/mL and a viability of 5.2 × 10^8^ CFU/mL, equal to 98%. Although Bioggp2 produced larger spores, its viability, and percentage of germination were lower than Bioggp5.

Table 5 shows some fungal characteristics based on their response to pH, temperature, and herbicides. All cellulolytic microfungi isolates are able to grow at a low pH (3, 4, and 5), which indicates that they tend to be acidotolerant. Meanwhile, they are also able to grow at a temperature of 30 °C. Their ability to grow decreases when the temperature is increased to 37 °C, and only Bioggp 3 isolates are able to grow at that temperature. It is interesting to note the fungal growth response when microfungi isolates are grown on media containing 1% herbicide. All isolates were able to grow on media containing 1% ametryne, except Bioggp 6, whereas all these cellulolytic isolates grew on 1% diuron herbicide.

The response of the growth of xylanolytic microfungi isolates to pH, temperature, and herbicide factors was also interesting (Table 6). Isolates grown at low pH (3, 4, and 5) indicate that they continue to grow well. They are also able to grow at a temperature of 30 °C and decrease their ability to grow when the temperature is raised to 37 °C. In addition, the isolates are also resistant to 1% herbicide, which is shown by their ability to keep growing well.

The growth responses of ligninolytic microfungi isolates to pH, temperature, and herbicide are shown in Table 7. These microfungi isolates (Bioggp 2 and 5) are able to grow in low acidity (pH 3, 4, and 5), but in this process, it was also seen that increasing the pH of the media increases the color intensity of guaiacol oxidation. This shows that if the conditions are more acidic, the guaiacol oxidation reaction decreases or vice versa. The growth response to temperature was also seen if these ligninolytic isolates are not very resistant at high temperatures, while the response to the herbicide shows that the isolates are resistant to 1% diuron and not to 1% ametryne.

**Figure 2:**
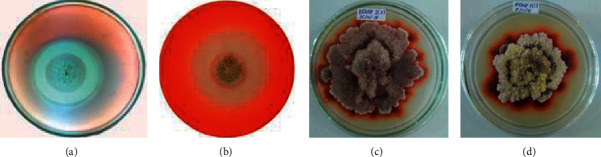
(a) Clear zone of cellulolytic (Bioggp 3); (b) xylanolytic (Bioggp 3) isolate; (c) oxidation of guaiacol of ligninolytic (Bioggp 2); and (d) (Bioggp 5) isolate on specific media.

The clear zone (halozone) that appears is a positive indicator that the isolates are able to degrade cellulose (CMC) (Figure 2). This is as explained by Shahriarinour et al. [21] that the clear zone formed is due to CMC which is the main carbon source in the growing media degraded by fungi. Cellulase is an enzyme complex consisting of cellobiohydrolase, endoglucanase, and *β*-glucosidase which synergistically propagate in degrading cellulose in nature. The clear zone around the fungi colony also occurs due to the isolate produce enzymes that are able to catalyze the xylan hydrolysis process. The xylan biodegradation process is a complex process which requires coordination of a number of xylanolytic enzymes hydrolyzing xylan and arabinoxylan polymers; among them are endo-*β*-1, 4- xylanase which attacks the main xylan chain and produces oligosaccharides [22]; and *β*- D-xylosidase which hydrolyses xylo-oligosaccharides to D-xylose [23]. Qualitative testing of the hydrolytic capacity (hydrolytic capacity) of each isolate was measured by comparing the ratio of the diameter or area of clear zones and their colonies [15].

The screening process for ligninolytic fungi is carried out by detecting the presence of brown color under and around the colony that indicates the presence of an oxidation reaction of lignin [6, 16]; the darker the color diameter which appears (dark brown) indicates that the lignin oxidation reaction by activity of the enzyme gets stronger. The guaiacol oxidation reaction is a test method, the most convincingly qualitative method used in the production of enzymes lignin degradation (lignin modifying enzymes (LMEs)) in microfungi.

## 4. Conclusions

Isolate Bioggp 3 has the highest cellulolytic and xylanolytic index among all isolates, although it does not correlate with the number of spores and percentage of spore viability. Isolate Bioggp 5 has the highest ligninolytic index and spore viability percentage. Most of the isolates were resistant to acidic conditions, could not withstand high temperatures, and were resistant to herbicides 1% (ametryne and diuron), except for ligninolytic isolates which could not stand ametryne 1%.

## Figures and Tables

**Table 1:** Isolation and enzymatic selection of fungi isolates from pineapple plantations.

No	Isolates	Cellulolytic	Xylanolytic	Ligninolytic	Genus

1	Bioggp 2	−	−	+	*Trichoderma* sp 1
2	Bioggp 3	++	++	−	*Aspergillus* sp. 1
3	Bioggp 4	−	−	−	—
4	Bioggp 5	−	−	+	*Trichoderma* sp 2
5	Bioggp 6	+	+	−	*Aspergillus* sp. 2
6	Bioggp 7	−	−	−	—
7	Bioggp 8	−	+	−	*Fusarium* sp
8	Bioggp 9	+	+	−	*Penicillium* sp.
9	Bioggp 10	−	−	−	—
10	Bioggp 11	+	−	−	Unidentified
11	Bioggp 12	+	+	−	*Paecilomyces* sp.

Note: + = enzymatic activity; − = no enzymatic activity.

**Table 2:** Cellulolytic index, spore numbers, and viability of isolates.

Isolates	Cellulolytic index	Spore numbers (spores/mL)	Spore viability (CFU/mL)	Percentage of spore viability (%)

Bioggp 3	4.00 ± 0.783^b^	2.0 × 10^8^	4.7 × 10^7^	91.5
Bioggp 6	1.26 ± 0.109^a^	1.5 × 10^8^	6.0 × 10^7^	95.1
Bioggp 9	1.17 ± 0.041^a^	4.5 × 10^8^	1.2 × 10^8^	93.3
Bioggp 11	1.09 ± 0.011^a^	1.7 × 10^8^	4.5 × 10^6^	80.8
Bioggp 12	1.52 ± 0.309^a^	2.7 × 10^8^	4.4 × 10^6^	78.7

Note: the data followed by the same letter indicates no significant differences.

**Table 3:** Xylanolitic index, spore numbers, and viability of isolates.

Isolates	Xylanolitic index	Spore numbers (spores/mL)	Spore viability (CFU/mL)	Percentage of spore viability (%)

Bioggp 3	4.20 ± 1.03^a^	2.5 × 10^9^	2.6 × 10^8^	89.3
Bioggp 6	1.90 ± 0.42^b^	2.5 × 10^8^	1.0 × 10^7^	83.3
Bioggp 8	1.20 ± 0.17^b^	1.8 × 10^7^	8.5 × 10^5^	80.8
Bioggp 9	1.50 ± 0.12^b^	4.6 × 10^9^	2.6 × 10^8^	86.6
Bioggp 12	1.20 ± 0.07^b^	2.2 × 10^9^	2.2 × 10^6^	67.7

Note: the data followed by the same letter indicates no significant differences.

**Table 4:** Ligninolytic index, spore numbers, and viability of isolates.

Isolates	Ligninolytic index	Intensity	Spore numbers (spores/mL)	Spore viability (CFU/mL)	Percentage of spore viability (%)

Bioggp 2	1.25 ± 0.06	3	1.8 × 10^9^	6.0 × 10^8^	95.0
Bioggp 5	1.47 ± 0.22	4	8.3 × 10^8^	5.2 × 10^8^	98.0

Remarks of scale of intensity: 4 = dark brown; 3 = brown; 2 = light brown; 1 = pale brown; and 0 = no color.

**Table 5:** The effect of pH, temperature, and herbicides on the growth of cellulolytic fungi.

Isolate	pH			Temperature (°C)			Herbicides (1%)	
	3	4	5	30	35	37	Ametryne	Diuron

Bioggp 3	+	+	+	+	+	+	+	+
Bioggp 6	+	+	+	+	+	−	−	+
Bioggp 9	+	+	+	+	−	−	+	+
Bioggp 11	+	+	+	+	−	−	+	+
Bioggp 12	+	+	+	+	+	−	+	+

Note: +: grow; −: not grow.

**Table 6:** The effect of pH, temperature and herbicides on the growth of xylanolytic fungi.

Isolate	pH			Temperature (°C)			Herbicides (1%)	
	3	4	5	30	35	37	Ametryne	Diuron

Bioggp 3	+	+	+	+	+	+	+	+
Bioggp 6	+	+	+	+	+	+	+	+
Bioggp 8	+	+	+	+	−	−	+	+
Bioggp 9	+	+	+	+	+	−	+	+
Bioggp 12	+	+	+	+	+	+	+	+

Note: +: grow; −: not grow.

**Table 7:** The effect of pH, temperature, and herbicides on the growth of ligninolytic fungi.

Isolate	pH						Temperature (°C)			Herbicides (1%)	
	Fungi growth			Color zone							
				(intensity)							
	3	4	5	3	4	5	30	35	37	Ametryne	Diuron

Bioggp 2	+	+	+	1	2	3	+	−	−	−	+
Bioggp 5	+	+	+	2	3	4	+	−	−	−	+

Remarks of scale of intensity: 4 = dark brown; 3 = brown; 2 = light brown; 1 = pale brown; and 0 = no color.

## Data Availability

The data are available from the corresponding author upon reasonable request.
